# Termites, hemimetabolous diploid white ants?

**DOI:** 10.1186/1742-9994-5-15

**Published:** 2008-09-29

**Authors:** Judith Korb

**Affiliations:** 1 Biologie I, University of Regensburg, D-93040 Regensburg, Germany; 2 Behavioral Biology, University of Osnabrück, D-D-49076 Osnabrück, Germany

## Abstract

Ants and termites are the most abundant animals on earth. Their ecological success is attributed to their social life. They live in colonies consisting of few reproducing individuals, while the large majority of colony members (workers/soldiers) forego reproduction at least temporarilly. Despite their apparent resemblance in social organisation, both groups evolved social life independently. Termites are basically social cockroaches, while ants evolved from predatory wasps. In this review, I will concentrate on termites with an ancestral life type, the wood-dwelling termites, to compare them with ants. Their different ancestries provided both groups with different life history pre-adaptations for social evolution. Like their closest relatives, the woodroaches, wood-dwelling termites live inside their food, a piece of wood. Thus, intensive costly food provisioning of their young is not necessary, especially as young instars are rather independent due to their hemimetabolous development. In contrast, ants are progressive food provisioners which have to care intensively for their helpless brood. Corresponding to the precocial – altricial analogy, helping by workers is selected in ants, while new evidence suggests that wood-dwelling termite workers are less engaged in brood care. Rather they seem to stay in the nest because there is generally low selection for dispersal. The nest presents a safe haven with no local resource competition as long as food is abundant (which is generally the case), while founding a new colony is very risky. Despite these differences between ants and termites, their common dwelling life style resulted in convergent evolution, especially winglessness, that probably accounts for the striking similarity between both groups. In ants, all workers are wingless and winglessness in sexuals evolved in several taxa as a derived trait. In wood-dwelling termites, workers are by default wingless as they are immatures. These immatures can develop into winged sexuals that disperse and found a new nest or into neotenic replacement reproductives that inherit the natal colony. Depending on the worker instar from which the latter develop, the neotenic reproductives are either apterous or brachypterous, but never winged. I propose that this wing polyphenism might present a basis for the evolution of social life in termites.

## Introduction

Termites (Isoptera) and social Hymenoptera (ants and some bees and wasps) are the classical social insects. They live in complex societies and are characterized by reproductive division of labour where only few individuals within a colony reproduce (in termites: usually a king and a queen; in social Hymenoptera: one or few queens) while the large majority of a colony forgoes own reproduction, at least temporarilly. How such altruistic behaviour can evolve under competition-driven Darwinian selection was one of the most intriguing problems in evolutionary biology. The answer central to this question is kin selection theory, i.e. the propagation of gene-coded traits via close relatives [1,2]. According to Hamilton's rule, altruism will be favored when *rb *> *c*, where *r *is the relatedness between the altruist and the recipient and *b *and *c *are the benefit and cost of the action to the altruist and recipient respectively [1].

Termites have often been compared with ants (for a recent comprehensive review see [3]). The termites' resemblance to ants, which is so striking that they are commonly referred to as 'white ants', lead to the assumption of convergent evolution driven by the same common factors. Indeed, more than 75% of all termite species (all higher termites, Termitidae) are characterized by altruistic castes (workers and soldiers) that help in raising offspring and have a greatly reduced capability to reproduce [4-6]. However, the Termitidae are a highly derived group and cannot be used to assess how eusociality may have first evolved within the termites. In fact, recent quantitative studies of species from families with a wood-dwelling life type that is thought to be ancestral in termite's evolution suggest that this does not apply to them (for recent discussions of termite phylogeny see [7,8]). Direct benefits of inheriting the natal nest rather than altruistic helping seem to be main selective forces for the occurrence of a defensive reproductive morph in dampwood termites [9] and for 'workers' in drywood termites [10] (see also [11]). This contrasts sharply with social Hymenoptera where altruistic helping was the major driving force (probably with the exception of some wasps) for the evolution of complex societies [12-14]. It is generally difficult to deduce the ancient evolutionary history from studies on extant species. Yet, the results for wood-dwelling termites may allow important conclusions because the wood-dwelling life type has idiosyncratic properties (e.g. poor nutritive quality of the food, bonanza type food resource) which *per se *set the selective environment for the evolution of cooperation and altruism in termites [15] (see also below). Therefore it seems reasonable to extrapolate the recent results to the evolutionary history and conclude that in termites costly altruistic helping by food provisioning probably only evolved after living in extended family groups [7,10] and that the initial step in termites' social evolution was characterized by immature offspring staying at home without intensive brood care of siblings [16].

For the reminder of this review, I will concentrate on wood-dwelling termites with an ancestral life type to compare them with social Hymenoptera, and especially ants. Occasional notes on other termite species will be given, wherever appropriate. As recent evidence indicates that the workers of the wood-dwelling termite species do not seem to considerably invest in brood care, I will refer to them as 'false workers' hereafter [7]. This slightly out-fashioned term sets them apart from 'true workers' of non-wood-dwelling species (i.e. all other termites), which forage outside the nest and take intensive care for the young of their colony (foraging termites hereafter) [7]. The false workers have sometimes also been called pseudergates, but as this latter term has originally a more restrictive definition [5,17], it should be avoided [7]. False and true workers not only differ in their function but, probably not coincidentally, also in their developmental options. True workers have a reduced developmental flexibility as they cannot become winged sexuals (e.g. [5]) and caste determination might even have a genetic component [18]. False workers, on the contrary, are ontogenetically totipotent immatures that can develop into (i) sterile soldiers, (ii) winged dispersing sexuals that found a new colony, or (iii) apterous or brachypterous neotenic reproductives that inherit the natal breeding position without dispersal (e.g. [5,19]).

In this comparative review, I will address selected topics that are emerging from recent results on wood-dwelling termites. For more general comparisons between social Hymenoptera and termites, I refer to [3,20-22]. First, I will outline why termites are not just hemimetabolous diploid, white ants. Their different ancestries provided termites and ants/social Hymenoptera with different life history pre-adaptations for social evolution. They explain why wood-dwelling termites – unlike ants but probably like the termites' ancestors – apparently seem to invest little in raising offspring, and how the ancestors' life histories facilitated termites' social evolution. I propose the idea that wing polyphenism, present in hemimetabolous insects in general [23,24] and cockroaches in particular [25], builds the molecular ground plan for termites' sociality. Despite their different ancestry, termites and ants also share selective regimes and these might have resulted in convergent evolution of winglessness and social organisation found especially in higher termites.

### Different ancestors – different prerequistes for social life

Termites are the oldest social insects with their complex societies dating back at least to the early Cretaceous (140 Mio) when they had dinosaurs as their contempories [26]. They are basically social cockroaches [27-29]. Although their position was somewhat debated [30,31], the weight of evidence from molecular markers and morphological traits now strongly suggests that the termites form a monophyletic clade within the Blattodea, most likely being the sister group of the Cryptocercidae (woodroaches) [27,28]. Similar to wood-dwelling termites, representatives of the appropriately named, monogeneric woodroaches, live as family units inside logs where they are able to digest wood with the help of symbiotic gut symbionts [25,32,33].

By contrast the eusocial Hymenoptera evolved complex societies at least 11 times independently from different ancestors within the Hymenoptera [12,22,34]. Although morphological, molecular, paleontological studies have presented conflicting views on ants early evolution [12,35,36], they certainly evolved from predatory wasps [12].

#### Need to help: A precocial – altricial analogy and its implications

The wood-dwelling termites are very unusual among social insects in that there are low incentives to help as their young are rather precocial. As hemimetabolous insects the young instars are quite independent. They can move around and do not rely on intensive brood care [10]. Especially, as the colony lives inside its food, there is no necessity for costly foraging and food is easily accessible to all individuals [16]. Thus, individuals only need to be infested with gut symbionts to exploit the common wood resource and these reciprocal infestations are less costly. As a consequence, the older offspring have few opportunities to reduce the work load of reproductives and hence can hardly gain indirect benefits by raising siblings [15,16]. Correspondingly, the annual growth rates of wood-dwelling termite colonies are quintessentially like those of solitary insects. Only 20 to 100 offspring per year are produced [21].

All this changes with a transition to a life style where individuals forage outside the nest, i.e. with the transition to foraging termites. Although many of these species still nest in dead wood, they all forage outside for additional food. In foraging termites, young depend on brood care by food provisioning [37]. This can be handed over from parents to older offspring (which become true workers) and the latter can further reduce the work load of reproductives by providing them with food as well [38,39]. So reproductives can concentrate and specialize on reproduction and annual colony growth rates increase reaching extreme values in fungus-growing termites with queens laying up to 40,000 eggs daily [37,40]. Accompanying specialization on reproduction, true physogastry (i.e., the enlargement of the abdomen through an increase in the number of functional ovarioles and fat bodies) evolved in queens of foraging termites [41]. On the other side, true termite workers evolved morphological adaptations to foraging and food provisioning [39]. For instance, sclerotization of the cuticle, which is largely absent in false workers, protects true workers against their hostile foraging environment. However, this comes with a cost as it hinders subsequent molts and thus prevents further development [5].

Wood-dwelling termites can also be further distinguished. They can inhabit two distinct nesting environments which might exert somewhat different selective pressures on social evolution: drywood termites (Kalotermitidae) generally nest in sound wood, while soft wood nesters (most dampwood termites, Termopsidae) occupy nests that are at least partially decayed by fungus [5]. In the former, the nest is a solid rather parasite- and pathogen-free environment, while the nests of dampwood termites are afflicted by high microbial loads [42]. Although data are scare so far and data for more species need to be collected, available evidence indicates that this might have consequences for brood care. In the drywood termites *Cryptotermes secundus*, *Cryptotermes cynocephalus *and *Cryptotermes domesticus *false workers do not care for eggs and young at all [10] (unpubl. data). From the second instar larvae onward individuals were seen to feed themselves. Eggs and first instar larvae are not cared for, they are not carried around, piled up or licked; but also they do not grow obviously until the next molt, which might suggest that they utilize body reserves. Similar data for the dampwood termite *Zootermopsis angusticollis *indicate that the brood is allogroomed (although other brood care like feeding is also absent) [43]. Further studies are clearly needed to illucidate whether this difference in grooming behaviour and pathogen load between damp- and drywood termites proves to be consistent. Based on the more basal phylogenetic position of the dampwood termites [4,8,29] and the fact that the termites' sister taxon, the woodroaches, inhabit damp wood [25,27], allogrooming to remove pathogen might have been the first and only component of brood care in the early evolution of termite's sociality that was already present in the termite's ancestors.

In contrast to the termites, social Hymenoptera are holometabolous insects. Their young are altricial, grub-like larvae which strongly depend on brood care, especially as social Hymenoptera always evolved from species with progressive food provisioning of the progeny [13,14,44]. This provides ample opportunities for alloparental care. Most modern ants (and many predatory wasps) have adults subsisting mostly on floral nectar or hemiptera exudates while hunting prey (or carrion) for the young [12]. As progressive food provisioners the adult female offspring can considerable reduce the work load of their mothers by foraging to feed their younger siblings (and their mother) so that mothers can concentrate on egg-production. Thus, ant workers can gain important indirect fitness benefits by specializing in the most costly investment in altricial young: food provisioning.

#### Possibility to stay: The importance of the food source

Another idiosyncratic property of wood-dwelling termites (and their ancestors) that largely distinguishes them from social Hymenoptera is their bonanza type food resource. Similarly, soil fed by soil feeding termites seems to constitute a bonanza food source but in contrast to wood it is too nutrient poor to live in it and grow to maturity. The nest of wood-dwelling termites, however, constitutes a resource that generally largely outlasts the lifetime of the founding primary reproductives. Hence offspring can stay with their parents as there is no local resource competition which normally selects for offspring dispersal [45]. Only when the wood block becomes exploited, resource competition shifts from a global (competition between colonies) to a local scale (competition among colony members) [46,47] and in line with this under limited food conditions, false workers develop into winged sexuals that leave the nest during the annual nuptial flight to found new colonies [48-52]. This is possible because the termites can sense food availability via vibrations generated during gnawing that act as reliable indicators of wood size [53]. During the developmental period from false worker via several nymphal instars (instars with wing buds) to winged sexual, which lasts several months [19], individuals of the drywood termite *Cryptotermes secundus *behave increasingly competitive. The degree of reciprocal proctodeal trophallaxis (= anal feeding) among false workers declines and each individual spends more time feeding wood [54]. Additionally, when proctodeal trophallaxis occurs it is preferentially directed at closer kin showing conditional nepotism in this species [55].

All this contrasts with most social Hymenoptera/ants which do not inhabit a bonanza type food resource. Social Hymenoptera seem to overcome local resource competition within a colony – and thus selection for dispersal – by increasing food intake through increased numbers of foragers [44]. 'Pay to stay' theory predicts that when staying of individuals poses costs to the dominant breeder rent payment can be selected [56,57]. Subordinate individuals (offspring, workers) might have to pay, for example in the form of foraging, in order to be allowed to stay in the colony [56,57]. Thus, helping to raise offspring might to some degree in some social Hymenoptera be a rent payment. This might especially apply to wasps or pleometropic foundress associations [58] where adult females face a threat of eviction but gain from staying in a group and where subordinates/helpers are not/less related to raised offspring.

Although there is no local resource competition in wood-dwelling termites when food is abundant, offspring competition can arise over reproductive inheritance, when the colonies' reproductives become unhealthy or die [59-62]. Then false workers which can develop into neotenic replacement reproductives compete for inheritance of the breeding position. In drywood termites such competition comes about in two manifestations [49,59]: (a) several false workers become neotenic replacement reproductives and they fight each other until one pair of reproductives is left; (b) only one replacement reproductive develops that immediately seems to inhibit the development of other false workers into replacement reproductives. Which individual will become the heir is apparently determined in a 'fair' process that is stable against cheating: During the intermolt period (note, false workers are larvae) there seems to be a very short phase of competence during which false workers are sensitive to the absence of reproductives [63,64]. As all false workers pass through this period, but only one or a few individuals (depending on colony size) are competent at any given time, each individual has a fair chance to inherit the breeding position.

This situation of local competition over inheritance might also counterselect altruistic helping and thus might further explain why false workers do not help in raising siblings. Taylor [46] and West and co-workers [65] have shown that under conditions of intense local competition (local competition = 1, global competition = 0) between relatives, as it occurs during the inheritance process, the benefits of altruism and the costs of competition cancel out each other so that altruistic helping will not be selected. Thus even though sharing resources with relatives is not costly because food is abundant, individuals should not invest in raising their strongest future competitors over the breeding position. The competition model further predicts that if intense local competition occurs among non-relatives spiteful behaviour might even be selected. Interestingly, in Cryptotermes interactions among non-relatives occur when two unrelated colonies that were independently founded in the same tree fuse during colony expansion [66]. It will be interesting to test whether non-relatives behave spitefully under such conditions.

#### The mode of sex determination and its consequences

Due to their different ancestry, termites and social Hymenoptera have different modes of sex determination. Social Hymenoptera are haplodiploid. Males develop from unfertilized eggs and are haploid, while females develop from fertilized diploid eggs. In contrast, termites are diploid with both sexes developing from fertilized diploid eggs. This results in symmetrical relatedness associations within monogamous termite colonies where the relatedness among fullsiblings and between parents and their offspring are identical (always r = 0.5). By contrast, in Hymenoptera fullsisters are more closely related to each other (r = 0.75) than parents are to their offspring (r = 0.5) or sisters are to their brothers (r = 0.25). This has important consequences: First, the unusually high relatedness between fullsisters in social Hymenoptera was initially thought to explain the multiple origins of eusociality in this order and the female preponderance in these colonies ([1,67]; for more recent discussions: [22,34,68]). However this haplodiploidy hypothesis faded as it became clear that haplodiploidy will only promote altruism relative to diploidy under rather restricted conditions [68]. Second, haplodiploidy creates relatedness asymmetries which result in conflicts between queens and workers over the sex ratio of sexuals and male production [69]. Similar conflicts are lacking in termites [61]. Third, Teyssèdre and co-workers [70] recently showed that the spread of altruism in diploids requires a pleiotropic link between altruism and enhanced productivity. By contrast, in haplodiploid organisms altruism within families that even lowers the productivity may spread, provided daughters sacrifice their own reproduction to raise full-sisters. A link between altruism and enhanced colony efficiency is unlikely when a mutant causing altruism occurs in a solitary organism. However, if such an altruistic mutant evolves in an already established group of coexisting relatives than it could automatically increase group productivity. This might have actually happened during the evolution of termites' eusociality [16]. As recent results indicate for wood-dwelling termites, in the termites' ancestors offspring probably stayed as false workers in the nest that did not invest more in raising siblings than woodroaches. So, family groups formed in which only in a second step an altruist mutant, the soldier caste, evolved. By defending the colony they considerably increased the group's reproductive success. Thus, altruism would be linked to enhanced productivity because large, less altruistic family groups already existed.

### Common selective regimes – convergent evolution: Winglessness

**Figure 1:**
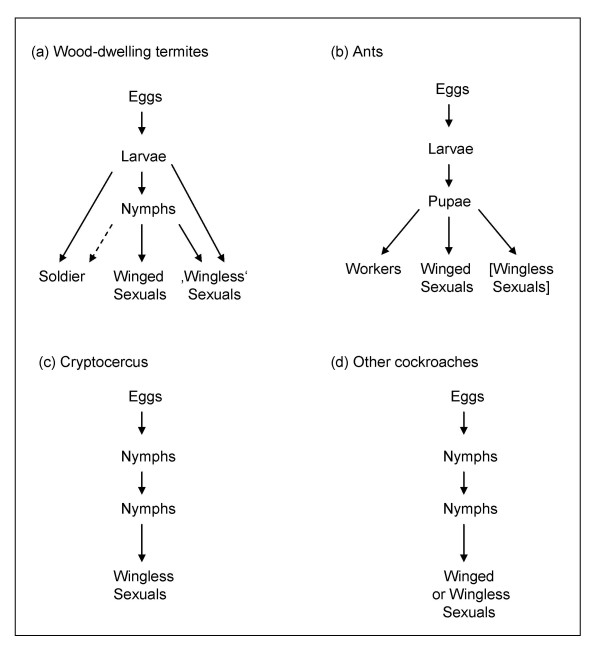
**Schematic development of (a) wood-dwelling termites, (b) ants, (c) the wood-roach Cryptocercus, and (d) other cockroaches, with special emphasis on wing-occurrence.** Shown are simplified diagrams of development, single instars are not shown. In wood-dwelling termites, 'wingless' sexuals are neotenic reproductives that can be either apterous or brachypterous depending on the instar from which they developed (see text). In ants, there generally is a bifurcation of development. At latest at the pupal instar (but often much earlier), individuals are determined to become either workers or winged sexuals, or in few cases wingless sexuals. In 'other cockroaches' wing dimorphism is often a sexual dimorphism. Note, the larvae of ants and termites are not equivalent. In termite terminology, instars without wing buds are called larvae, while those with wing buds are termed nymphs. In all other hemimetabolous insects (including the cockroaches) all instars are called nymphs.

Even though termites are phylogenetically very distant from ants, they share characters as result of common selective regimes that explain their striking resemblance. Both ants and termites have clearly evolved (independently) from winged solitary or primitively social ancestors [71], and winglessness is an adaptation to burrowing activities (Fig. 1). Crozier [12] speculated that dispersing, mating and settling on the ground predisposed such insects to form small family groups, leading naturally to a strong influence of kin selection. This fostered the further transition to the differentiation between queens and female workers in ants, and reproductives and soldiers of both sexes in termites.

Stemming from predatory wasps, ants have reduced the winged stage to a dispersal phase and adapted to life on or in the ground by females casting off their wings once they have mated. Some species went even further. As alternative reproductive strategies, they evolved wingless queens and males (i.e. sexual morphs) that reproduce within the natal nest or disperse by foot [72,73]. Thus, in ants the following castes can be found in different species: winged male and female sexuals, wingless males and female sexuals, and wingless (female) workers (Fig. 1).

Due to their hemimetabolic mode of development, the false workers of termites (males and females) are immatures and by default never have completely developed wings. Depending on the instar, they can have more and less developed wing buds. In contrast to ants, wings develop gradually and instars without wing buds are called larvae, while nymphal instars have wing buds [5,39,74]. The number of nymphal instars is highly variable and even species with a single nymphal instar exists [5,39,75,76]. Among the terminal instars that present an endpoint in development, soldiers are always wingless, while the dispersing sexuals are winged. Neotenic replacement reproductives that develop via a single molt from false workers, can be either apterous or brachypterous depending on the instar from which they developed (Fig. 1). Thus, the winged sexuals and the neotenic reproductives can be regarded as two morphs of a wing polyphenism that present two alternative reproductive tactics [77]. Similar (environmental induced) wing polyphenisms or (genetically based) polymorphisms can be found in other hemimetabolous insects, such as crickets or aphids (e.g. [23,24,78,79]) (Fig. 1). Interestingly, the triggers (e.g. group size/density, food availability, parasite load) inducing wing polyphenism are similar in these groups and the wood-dwelling termites (e.g. [23,77,78,80]).

As discussed above, direct fitness benefits gained by inheriting the natal breeding position as neotenic replacement reproductive seem to play an important role in wood-dwelling termites, and probably also during the termites' social evolution [7,11,16]. This might indicate that the genetic architecture underlying wing polyphenism in solitary hemimetabolous insects builds the molecular ground plan from which termites' sociality developed.

Interestingly, wing polymorphism is common in cockroaches [25]. Winglessness is an evolutionarily labile trait that occurs in all taxonomic groups [25,81]. Although macropterism clearly is the primitive condition, for instance in the Panesthiinae apterous, brachypterous and species that loose their wings (macropterous, deciduous) seem to have evolved several times [25]. Cockroaches that spend their entire life in burrows, galleries, or crevices, except for a brief dispersal period, seem most prone to winglessness. Three characteristics of crevices and burrows have been proposed to influence wing loss in cockroaches [25]: First, they are homogeneous microhabitats, in that they are interchangeable dark, moist, protected quarters. Second, these are chiefly two-dimensional microhabitats lacking space for flight. Third, they are temporally stable habitats where food (e.g. logs, leaf litter or other rotting vegetable material) is continuously or periodically replenished, and the cockroaches are able to feed within their shelter where they rely on low quality food. These idiosyncratic characteristics are similar to those found in wood-dwelling termites [82]. Adults in the sistertaxon of the termites, the Cryptocercus woodroaches are wingless [32]. This might indicate that the molecular basis for wing polymorphism was most probably already present in the termites' ancestors with termites exploiting it during the evolution of two alternative breeding tactics, winged dispersing sexuals that found new colonies and wingless neotenic sexuals that become natal reproductives. In contrast, woodroaches became completely wingless.

Aptery and brachyptery are generally associated with a developmental syndrome that reduces complexity (e.g.[25]). They are the best indicator of paedomorphosis, defined as the retention of juvenile characters of ancestral forms in the adults of their descendents. Similarily, the neotenic reproductives of termites lack so-called 'adults characters' such as compound eyes or antennal segments, suggesting that they are a result of paedomorphosis [30] and thus of a heterochronic shift of gene expression (see also [83]). As a comparison, in the best studied social insect, the honeybee *Apis mellifera*, adult workers assume the worker role through a shift in expression of maternal care genes before foraging (e.g. [84-86]). The expression of genes for maternal care is rerouted to precede foraging, thus reversing the normal sequence in adult developmental ground plan of the ancestors.

Altogether, these results suggest that based on wing polymorphisms different reproductive tactics exist in both wood-dwelling termites and ants. However, while wing polyphenism seems to build the basis in termites' social evolution and is lost in derived species which only reproduce via winged sexuals [77], wing polyphenism as an alternative reproductive tactic seems to be a derived trait in ants. Whether the same genes are involved in wing polyphenism in ants and termites remains to be tested. Judging from the phylogenetically well-separated flies (order Diptera) and butterflies (order Lepidoptera), wing development seems to be directed throughout the winged insects by an unchanged regulatory gene network and not only the individual signaling pathway elements, but even entire gene regulatory networks turned out to be highly conserved across species, orders and even phyla [87]. The *Drosophila *wing formation network has been successfully employed in a study on the loss of wings in workers of several ant species [88].

## Conclusion

In contrast to ants where workers gain considerable indirect fitness benefits through provisioning of progeny, the false workers of wood-dwelling termites seem to be less altruistic with regard to brood care. In terms of Hamilton's rule, this can be explained by low benefits to gain through food provisioning of young as they are hemimetabolous insects that nest inside their food. At the same time, the costs of staying at the nest are low for wood-dwelling termites. Staying does not reduce their direct fitness prospects, but rather increases them: False workers can wait in a safe haven, protected against hostile environmental conditions, until they have aquired enough resources to disperse or inherit the natal breeding position when the same sex reproductive of their colony dies. Staying at the nest also has no negative consequences for relatives (i.e. no negative indirect fitness consequences) as long as food is abundant and the current reproductives are healthy. The prospect of future local competition might even select against raising siblings which become potential competitors. These idiosyncratic properties of a wood-dwelling life style inherited from a cockroach ancestor, set a completely different selective regime than that which was present in the predatory ancestors of ants. Nevertheless, the social, ground-dwelling life style of ants also selected for a wingless life which was present in termites before they become eusocial. This common selective environment of 'dwelling in the dark' probably accounts for the striking resemblance between ants and termites and their social organisation.

## Competing interests

The author declares that she has no competing interests.
